# A Novel Storm Cell Identification, Tracking and Nowcasting Algorithm for Severe Thunderstorm Winds Using Networked Weather Radar Data

**DOI:** 10.3390/s26217027

**Published:** 2026-11-06

**Authors:** Yuanbo Ran, Haijiang Wang, Taofeng Gu, Fang Liu, Bo Zhang

**Affiliations:** 1 Fuling District Confidential Technology Service Center, Chongqing 408000, China; ryb1134471679@163.com; 2 College of Electronic Engineering, Chengdu University of Information Technology, Chengdu 610225, China; whj@cuit.edu.cn; 3 Guangzhou Meteorological Comprehensive Guarantee Center, Guangzhou 511430, China; 4 Chongqing Meteorological Data Center, Chongqing 401147, China; cmaliufang@126.com; 5 Shenzhen Institute for Advanced Study, University of Electronic Science and Technology of China, Shenzhen 518000, China

**Keywords:** severe thunderstorm winds, storm cell, identification, tracking, nowcasting, fuzzy logic, unscented Kalman filter, adaptive damped holt

## Abstract

As a prevalent meteorological hazard across China, severe thunderstorm winds originate directly from storm cells. With the dense networking of Doppler weather radars in recent years, networked radar observations have become an essential technical support for the operational monitoring and early warning of severe thunderstorm winds. Traditional storm cell identification, tracking and nowcasting methods are mainly tailored to single-radar datasets. To overcome the limitations of traditional methods, this study proposes a novel method for storm cell identification, tracking, and nowcasting based on networked radar data. The method comprises three steps: identifying 3D storm cells via digital image processing and assessment their intensity using fuzzy logic; constructing a minimum cost function for spatiotemporal matching and continuous tracking; and finally, adopting the unscented Kalman filter and the adaptive damped Holt method to forecast the future trajectory and intensity, respectively. Compared with the traditional operational nowcasting methods adopted by meteorological services in China, namely storm cell identification and tracking (SCIT) and thunderstorm identification, tracking, analysis and nowcasting (TITAN), the proposed method exhibits growing advantages in storm location prediction within the 60-min forecast lead time, with controllable decay of probability of detection (POD) and critical success index (CSI) and low false alarm rate (FAR). The developed intensity forecast product enriches the storm forecasting framework, with its mean absolute error (MAE) kept within a controllable range to support operational short-range nowcasting.

## 1. Introduction

Severe thunderstorm winds are prevalent meteorological hazards across China, characterized by small spatial scales, intense wind speeds, and rapid genesis and dissipation [1,2]. They impose great difficulties on operational forecasting and early warning systems. As defined by the China Meteorological Administration (CMA), severe thunderstorm winds are strong winds occurring under severe convective weather with instantaneous wind speeds above 17.2 m s^−1^, which inflict severe damage on industry, agriculture, transportation and public livelihood.

Doppler weather radar is an active remote-sensing sensor widely used for meteorological observation. It transmits electromagnetic waves into the atmosphere; hydrometeors scatter these waves, and the radar receives the backscattered echoes. After signal processing, cloud and storm information within the atmosphere can be retrieved. With the dense networking of Doppler weather radars in recent years, networked radar observations have become an essential technical support for operational monitoring and early warning of severe convective weather. Doppler radar data feature high temporal and spatial resolutions. Through identification, analysis and feature tracking of radar echoes, the evolutionary trends of heavy rainfall weather systems can be captured intuitively and rapidly. Nowcasting of severe convective weather based on radar echo extrapolation hinges on fully exploiting the temporal continuity and spatial correlation of volume scan data. Interpolation reconstruction, adaptive filtering and spatial smoothing are performed on the reflectivity factor field to extract the advection motion field of echo systems as well as their morphological evolution tendencies [3,4]. These outputs are then used to drive an extrapolation model for quantitative prediction of precipitation intensity and distribution over the next several hours.

Currently, SCIT and TITAN are two of the most widely adopted and technically mature algorithms operationalized for convective weather nowcasting in China. The SCIT algorithm traverses radar base data with multiple reflectivity thresholds to identify storm segments along radar radials. Two-dimensional storm cells are constructed by merging adjacent azimuthal storm segments, and three-dimensional storm structures are further formed by integrating vertically adjacent and spatially overlapping two-dimensional cells. Key storm characteristics, including centroid position, volume, and projected area, are extracted to match and track convective cells across successive radar volume scans. The mean storm motion vector is calculated based on centroid displacements during consecutive scanning periods, and future storm positions are predicted via linear extrapolation [5]. The SCIT algorithm achieves stable identification and tracking performance for conventional convective storms and satisfies routine operational nowcasting requirements. Nevertheless, its forecasting accuracy degrades rapidly with increasing lead time for rapidly evolving, genesis-dissipating convective systems with nonlinear motion characteristics.

Different from the multi-threshold scheme of SCIT, the TITAN algorithm treats precipitation cloud clusters as integral three-dimensional structures and identifies individual storm cells using a fixed reflectivity threshold of 30 dBZ, where contiguous echo regions exceeding the threshold are defined as independent storm units. Storm tracking is implemented based on the spatial distance and volumetric similarity of storm cells between adjacent observations. Temporal sequences of critical storm parameters, including movement velocity, coverage area, volume, and maximum reflectivity, are fitted using a weighted linear method to extrapolate future storm evolution. Meanwhile, the forecasting model adaptively adjusts the historical evolution sequences when storm merging or splitting occurs during the extrapolation process. Benefiting from its concise principles and high computational efficiency, TITAN is well suited for real-time meteorological operational applications [6]. However, this method fails to accurately characterize the genesis, dissipation, and nonlinear evolution of storm cells, leading to progressively growing forecasting errors with extended lead time under complex convective conditions involving superimposed storms and intense storm evolution.

In recent years, optical flow methods and deep learning approaches have been applied to radar echo extrapolation and achieved favorable forecasting performance. The optical flow method establishes inter-frame correspondence by exploiting the data correlation between radar echo images at adjacent moments, thereby calculating the displacement vector field of radar echoes [7]. Based on this fundamental principle, the Lucas-Kanade and Farneback optical flow algorithms have been developed as typical classical variants [8,9,10]. Furthermore, Novel optical flow methods including DeepFlow and PCAFlow have emerged to realize the adaptive adjustment of optical flow parameters [11,12]. These advanced approaches achieve substantial performance improvements in terms of spatial smoothness, multi-scale processing capability, and spatiotemporal consistency, effectively overcoming the limitations of traditional optical flow algorithms. Despite the excellent performance of the optical flow method in terms of accuracy and timeliness for short-term radar echo extrapolation, its application is constrained by the inherent brightness constancy assumption. For rapidly evolving severe convective weather systems, the radar reflectivity is non-conservative during the evolutionary process of weather phenomena. This fundamental inconsistency introduces substantial forecasting errors and severely degrades the predictive capability of the optical flow method for severe convective events.

Deep learning-based radar echo extrapolation models take sequential radar echo data as inputs. Through iterative training on massive datasets, these models automatically extract the spatiotemporal features and evolutionary patterns of radar echoes, thereby achieving accurate predictions of future radar echo fields. Classical deep learning frameworks for radar nowcasting mainly include convolutional neural networks (CNNs) and recurrent neural networks (RNNs), as well as their variants such as long short-term memory (LSTM) and gated recurrent unit (GRU) [13,14,15,16,17,18,19]. These architectures can effectively capture the spatiotemporal correlation of radar echoes and improve the accuracy and stability of short-term extrapolation. Nevertheless, most conventional deep learning models adopt grayscale images converted from pseudo-color radar echo data as inputs, which inevitably causes the loss of refined echo feature information and limits detailed convective prediction performance. In recent years, a series of representative studies published from 2022 to 2025 have promoted the refinement and optimization of radar nowcasting techniques. The multi-scale transformer-based MS-RadarFormer is proposed to capture complex spatiotemporal variations of radar echoes and improve extrapolation accuracy [20]. A spatiotemporal transformer network is designed to model dynamic echo evolution characteristics for reliable radar nowcasting [21]. Furthermore, an event-guided spatiotemporal transformer combined with conditional diffusion refinement achieves high-precision nowcasting of intense precipitation convection [22]. Despite their promising performance in full-field radar echo prediction, these state-of-the-art generative models generally suffer from high computational consumption and focus on overall grid echo field prediction, lacking targeted identification, tracking, and intensity evolution modeling for individual storm cells. Furthermore, the initiation and evolution of severe convective weather are strongly modulated by underlying terrain conditions. Since the performance of deep learning models relies heavily on massive historical training data, complex terrain can significantly reshape radar echo spatiotemporal evolution characteristics and form region-specific variation patterns. The existing models, whether traditional CNN/RNN frameworks or latest transformer and diffusion-based methods, still struggle to balance prediction accuracy and regional generalization ability, restricting their practical application in operational severe convective weather nowcasting.

Based on operational observations from China’s network radar system and the characteristic properties of constant altitude plan position indicator (CAPPI) products, this study develops a novel integrated framework for storm cell identification, tracking and nowcasting. The proposed method outperforms conventional algorithms remarkably in both prediction accuracy and operational practicability. This method makes the following contributions:

(1) We preprocess CAPPI gridded reflectivity products via morphological erosion and dilation operations to suppress radar clutter and eliminate missing-data artifacts. Contour detection is subsequently applied to segment and identify individual storm cells. The grid-based matrix computation adopted in this study ensures high recognition accuracy, precise storm boundary localization, and excellent computational efficiency, making the method suitable for real-time operational nowcasting applications.
(2) We construct a minimum cost function based on the three-dimensional spatial characteristics of storm cells to realize optimal matching of storm cells at adjacent moments and accurately track their temporal motion trajectories. Meanwhile, we adopt a fuzzy logic algorithm to quantitatively evaluate the intensity of individual storm cells. Combined with historical trajectory data, the temporal variation of storm intensity can effectively reveal the initiation, development and dissipation trends of storm cells.
(3) We combine the historical motion trajectories and intensity assessment results of storm cells and introduce the unscented Kalman filter algorithm to predict the moving paths and spatial location of storm cells in the next 0–1 h. The nonlinear trajectory predictions match the actual evolution of convective systems very well. Meanwhile, we adopt the Adaptive Damped Holt model to forecast storm cell intensity over the 0–1 h lead time, which can better satisfy the operational demands of short-range nowcasting.

## 2. Data and Preprocessing

**Figure 1:**
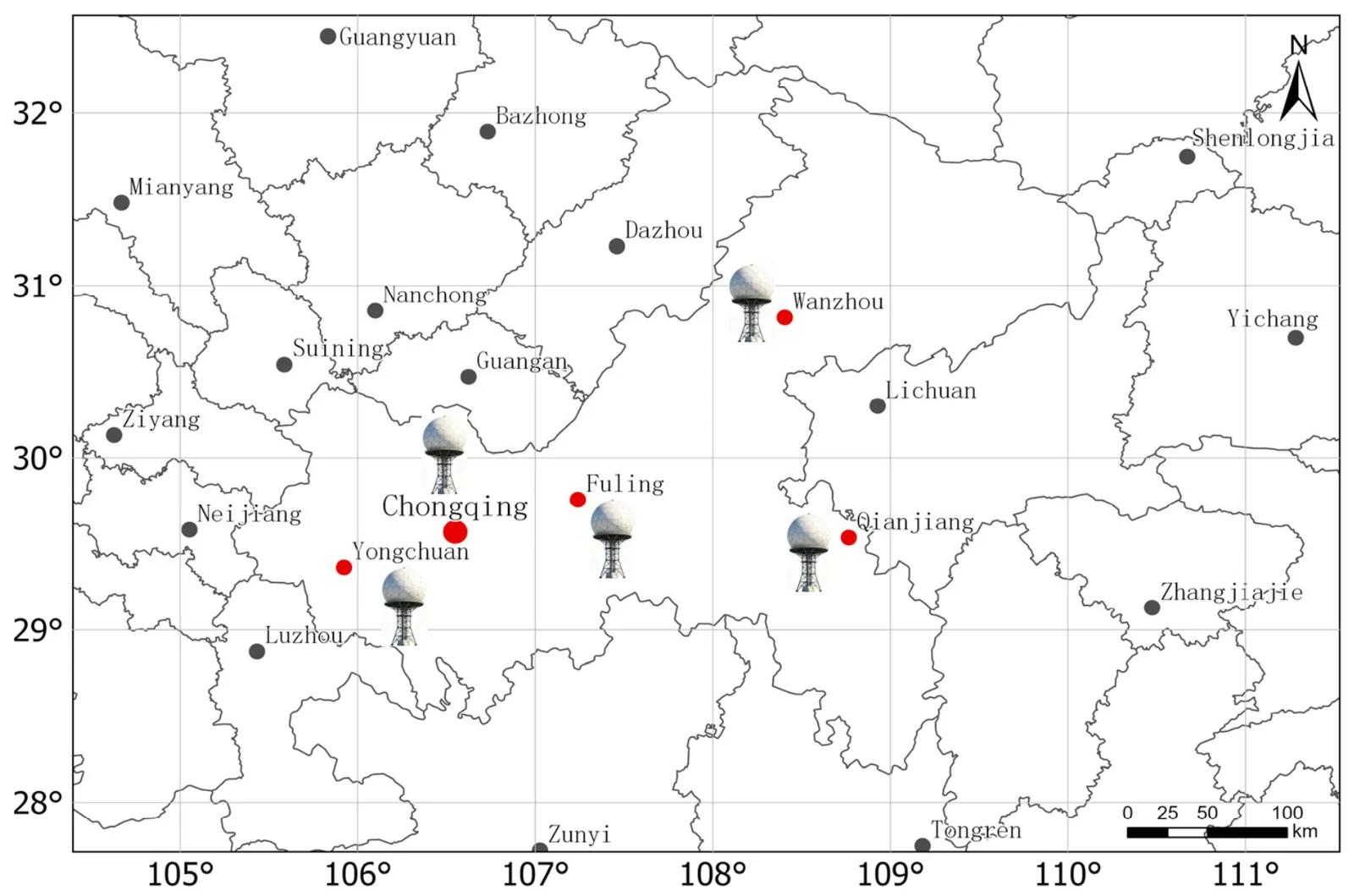
Distribution of Radar Stations for the Chongqing Networked Radar Observation System.

Chongqing, located in southwestern China, features predominant basin and mountainous terrain with the confluence of major rivers including the Yangtze, Jialing, and Wujiang Rivers. Complex topography triggers forced uplift of warm-moist air masses, establishing thermodynamically favorable atmospheric conditions for the genesis and development of severe convection. Accordingly, severe convective hazards are frequent across the region, with severe thunderstorm winds being one of the most common hazardous weather phenomena. The observational datasets used in this study were derived from the operational composite weather radar network of Chongqing. This network consists of five CINRAD-SA dual-polarization Doppler weather radars deployed in Chongqing, Wanzhou, Fuling, Yongchuan and Qianjiang. All radars are manufactured by Huayun Metstar Radar Co., Ltd., Beijing, China. Their spatial distribution and detailed information presented in Figure 1 and Table 1, respectively. Distributed across different districts of Chongqing, these five radars jointly form an integrated monitoring network that covers the entire Chongqing municipality and its surrounding vicinities. The composite radar products feature high spatial resolution and reliable detection accuracy.

All radars are sited at the highest elevation within their respective regions to ensure that the maximum blocked azimuth angle is ≤1° and the cumulative blocked azimuth angle is ≤2°. Adjacent radars have overlapping detection domains. For elevation layers affected by terrain occlusion, the networked radar data fusion system compensates observations by incorporating measurements from other radars, ensuring the integrity and reliability of the fused dataset and mitigating the impacts of terrain blockage.

The dataset utilized in this study is presented in Table 2, which contains observational records of 12 city-wide and 20 local severe thunderstorm wind events that occurred in Chongqing from 2023 to 2025. All collected cases were equally divided, where half of the samples (training cells) were employed for method parameter calibration, and the remaining half (testing cells) for quantitative model evaluation. Radar reflectivity products are available at five vertical levels (3 km, 4 km, 5 km, 6 km, 7 km) with a horizontal spatial resolution of 1 km × 1 km. Standardized preprocessing was implemented on the raw data prior to analysis. The processing workflow is described as follows. First, grid points with radar reflectivity below 10 dBZ were removed. Second, binary segmentation was applied to the CAPPI reflectivity fields at all vertical levels. Third, morphological operations consisting of erosion followed by dilation were performed sequentially to generate preprocessed CAPPI reflectivity datasets. This preprocessing scheme effectively eliminates discrete point clutter noise, connects adjacent isolated storm cells, and merges them into complete integrated storm entities.

## 3. Methods

**Figure 2:**
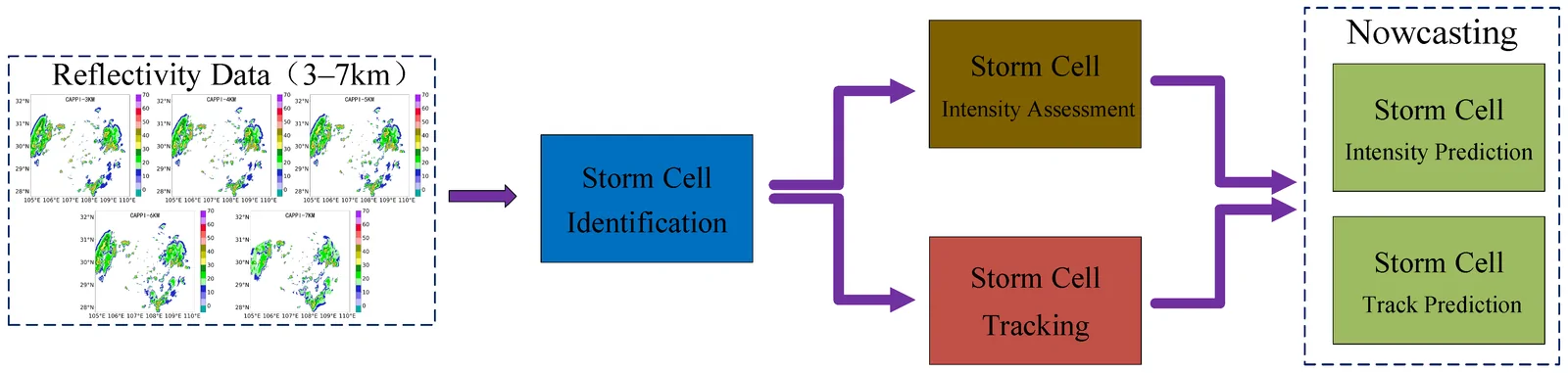
Overall Architecture for Identification, Tracking, Nowcasting of Storm cells.

The schematic flowchart of the proposed method is illustrated in Figure 2. The overall research framework consists of four core modules: storm cell identification, storm cell intensity assessment, storm cell tracking, and storm cell nowcasting. Specifically, the storm cell nowcasting module includes track prediction and intensity prediction.

### 3.1. Identification

Accurate identification of storm cells is essential for severe thunderstorm wind nowcasting and early warning operations. Based on the CAPPI features of networked radar observations and digital image processing techniques, this study utilizes the OpenCV built-in contour detection function findContours to automatically identify storm cells from CAPPI reflectivity fields within vertical layers of 3–7 km. The findContours algorithm is a classical and widely used contour detection method based on edge detection and topological analysis. It provides fundamental image processing functions, including contour rendering, contour area calculation, and point-in-contour judgment, which can support the contour extraction and quantitative characteristic analysis of storm cells [23].

Radar reflectivity thresholds of 30 dBZ, 40 dBZ and 50 dBZ were selected separately. The contour detection algorithm was applied to extract the contour boundaries of storm cells at each vertical level, yielding contour identification results of storm cells under multiple thresholds and vertical layers. Meanwhile, the centroid coordinates of storm cells within each contour were calculated, as shown in Equation (1), where (*x_i_*_,*j*_,*y_i_*_,*j*_) denotes the coordinates of grid cell (*i*,*j*),*Z_i_*_,*j*_ is its corresponding reflectivity (dBZ), and $\left(\bar{x},\bar{y}\right)$ is centroid coordinates of the storm cell.

(1) $$\bar{x}=\frac{\sum_{i}\sum_{j}Z_{i,j}x_{i,j}}{\sum_{i}\sum_{j}Z_{i,j}}\;\bar{y}=\frac{\sum_{x}\sum_{y}Z_{i,j}y_{i,j}}{\sum_{i}\sum_{j}Z_{i,j}}$$

**Figure 3:**
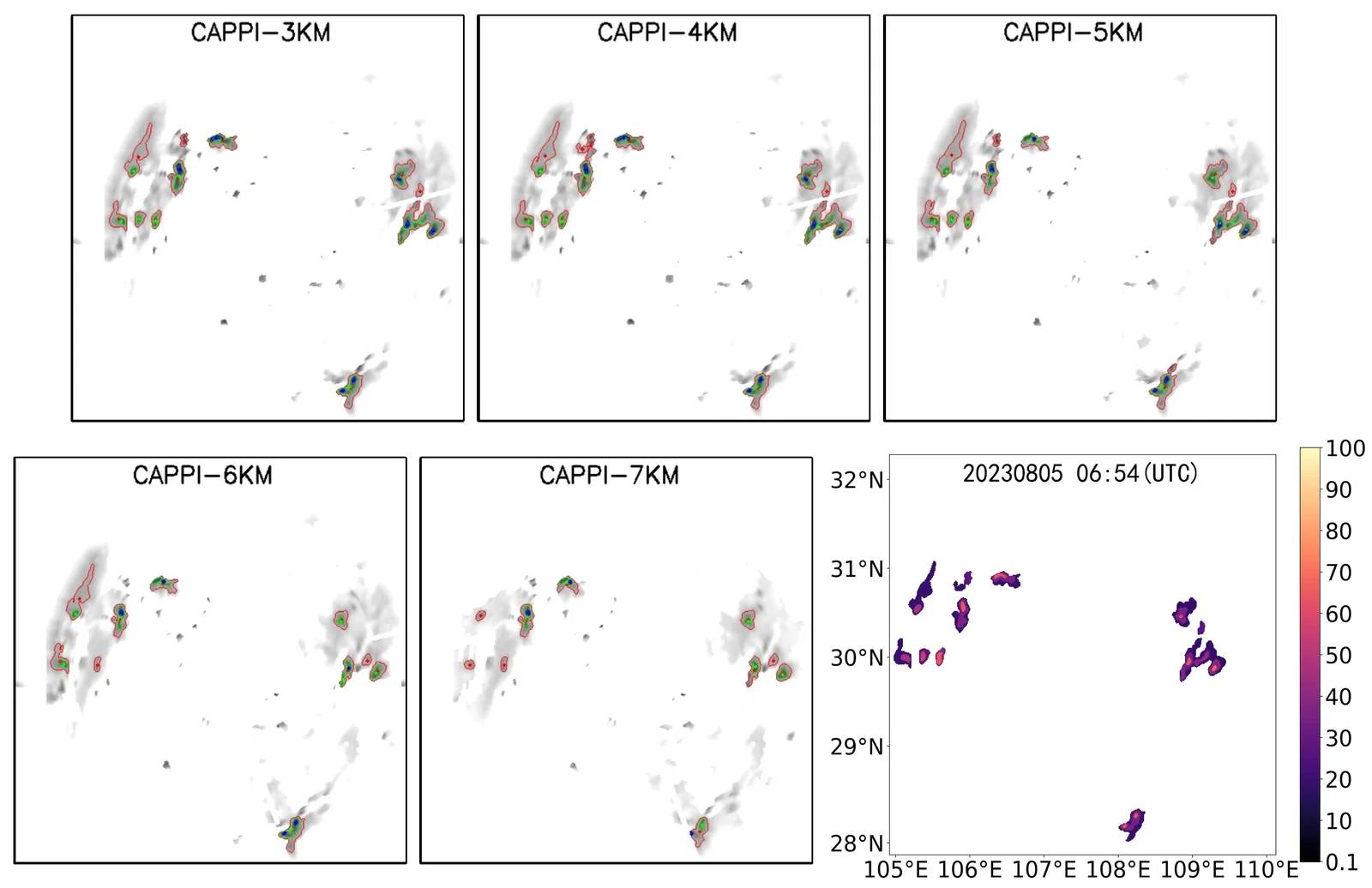
Schematic of storm cell identification.

Figure 3 illustrates the two-dimensional identification results of storm cells at all vertical layers. The red, blue, and green contours in the figure represent storm outlines derived from reflectivity thresholds of 30 dBZ, 40 dBZ, and 50 dBZ at each vertical level, respectively.

The association criteria for adjacent vertical layers are specified as follows:

(i) Two vertical layers are assigned to the same storm cell if the horizontal projection contours of their 30 dBZ echoes overlap.
(ii) Two layers are identified as the core region of a storm cell when the horizontal projections of their 40 dBZ echoes intersect.
(iii) With regard to storm cell centroids, two adjacent vertical layers are merged into a single cell group if either the horizontal projections of their 50 dBZ echoes overlap, or the horizontal distance between their respective centroids is ≤5 km.

### 3.2. Intensity Assessment

The horizontal scale of storm cells generally ranges from several kilometers to tens of kilometers, and mature storm cells can extend vertically to heights above 10 km. Based on evolutionary characteristics, the entire life cycle of storm cells is divided into three stages: initiation, intensification, and dissipation. In the initiation stage, compact discrete echo cells newly form with central reflectivity exceeding 30 dBZ, accompanied by rapid expansion of central intensity, horizontal coverage and vertical extent. During the intensification stage, the central reflectivity and horizontal span of cells increase continuously, while the echo top height rises rapidly to a maximum exceeding 10 km; meanwhile, the height of the three-dimensional storm centroid gradually lifts. In the dissipation stage, the central reflectivity weakens steadily and cell splitting frequently occurs, with synchronous declines in echo top height and three-dimensional centroid altitude. Using the aforementioned life-cycle characteristics of storm cells, we develop a fuzzy-logic storm cell intensity assessment model expressed in Equation (2).

(2) $$R=\sum_{i=1}^{3}w_{i}[\lambda_{i1}\mathrm{Trap}(x_{i1})+\lambda_{i2}\mathrm{Sig}(x_{i2})+\lambda_{i3}\mathrm{Gauss}(x_{i2})]$$

where *R* denotes the integrated intensity of storm cells derived from the model; the subscript *i* = 1,2,3 corresponds to three categories, namely the entire storm cell, storm cell core region, and storm cell centroid region, respectively. $\left[x_{i1},x_{i2},x_{i3}\right]$ represent the horizontal projected area, vertical extension height and centroid height of the given category, while *w*_1_ + *w*_2_ + *w*_3_ = 1 and *λ*_i1_ + *λ*_i2_ + *λ*_i3_ = 1 are weight coefficients. *Trap*(), *S*ig(), and *Gauss*() stand for three types of membership functions: trapezoidal, sigmoid, and Gaussian functions. The value of *R* ranges from 0.1 to 100, and a larger value indicates stronger storm cell intensity.

**Figure 4:**
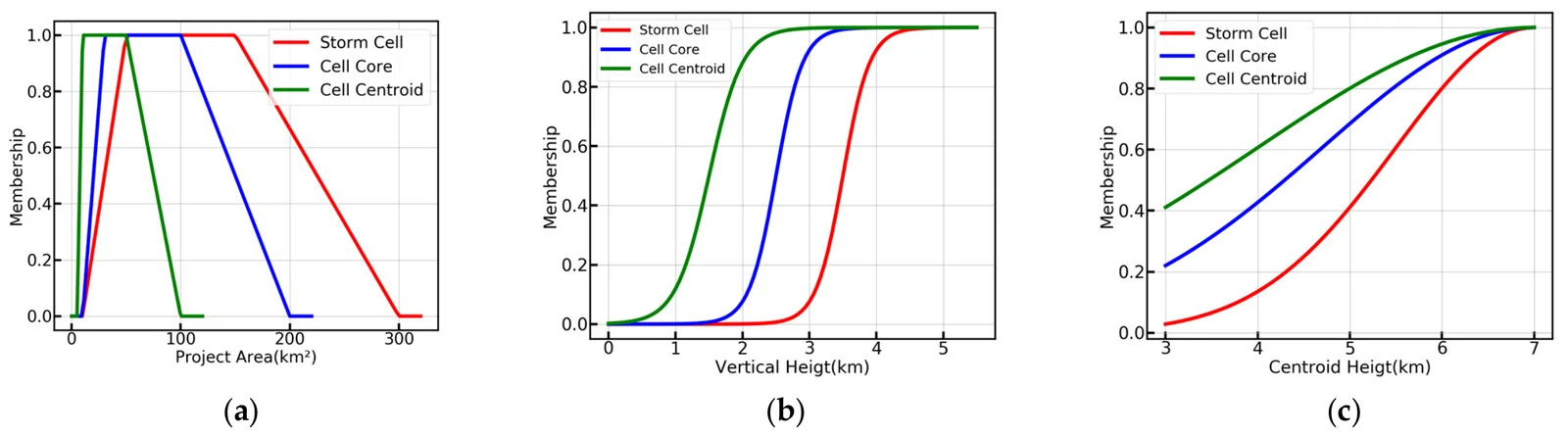
Distributions of (**a**) trapezoidal, (**b**) sigmoid, and (**c**) Gaussian membership functions.

The mathematical expressions and parameter settings of the trapezoidal, sigmoid, and Gaussian membership functions are listed in Table 3, and their distributions are displayed in Figure 4. Subplots (a), (b), and (c) correspond to trapezoidal, sigmoid, and Gaussian membership functions, respectively. The horizontal axes represent three storm cell features (horizontal projected area, vertical height, and centroid height), while the vertical axes denote the membership values of each feature. The red, green, and blue curves in each panel indicate the membership functions of the entire storm cell, storm cell core region, and storm cell centroid region for the assigned feature.

Figure 3 presents typical examples of storm cell intensity assessment results. Different color bars in the figure correspond to varying levels of storm cell intensity. All storm cells exhibit a consistent spatial intensity distribution, with the maximum intensity occurring in the core region and a gradual intensity attenuation from the core to the peripheral edges.

### 3.3. Tracking

**Figure 5:**
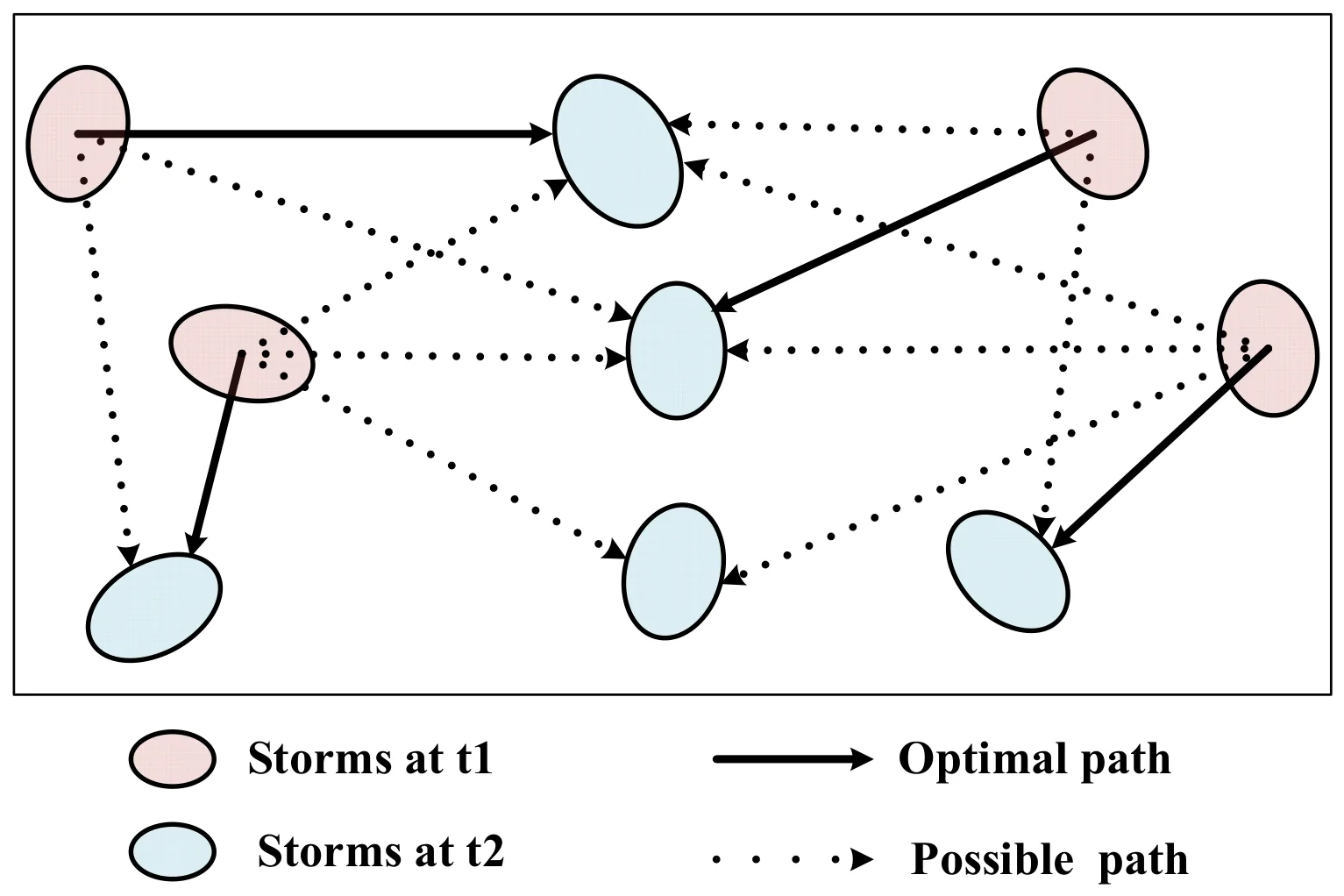
Schematic diagram of storm cell matching.

Doppler weather radars feature high temporal resolution, with a time interval of 5–6 min between successive scans. Restricted by the short sampling interval, storm cells generally exhibit small horizontal displacements and insignificant abrupt changes in shape and projected area across consecutive observations. The temporal matching principle of storm cells is demonstrated in Figure 5. During cell tracking, multiple candidate cells at time *t*_2_ can potentially match the target cell detected at time *t*_1_. Matching rule is established based on the spatio-temporal continuity of convective systems: the smaller the horizontal displacement and area variation rate between the candidate cell at time b and the original cell at time a, the higher the matching confidence that they belong to the same storm cell.

Based on the storm cell identification results obtained above, a cost function is constructed to match storm cells at adjacent times. The cell pair with the minimum cost function value is determined as the optimal match, which indicates that the two cells correspond to the same storm cell captured at different observation times. Assume that the state of the i-th storm cell at time *t*_1_ is denoted as $\psi_{1i}=[({\bar{x}}_{1i},{\bar{y}}_{1i}),s_{1i},(vx_{1i},vy_{1i})]$, and the state of the j-th candidate cell to be matched at the subsequent time *t*_2_ is $\psi_{2j}=[({\bar{x}}_{2j},{\bar{y}}_{2j}),S_{2j}]$. The cost function C between cell i and cell j is defined in Equation (3).

*C_ij_* = *w*_1_*d_p_* + *w*_2_*d_s_* (3)

(4) $$d_{p}={\left[{\left({\bar{x}}_{1i}-{\bar{x}}_{2j}\right)}^{2}+{\left({\bar{y}}_{1i}-{\bar{y}}_{2j}\right)}^{2}\right]}^{1/2}$$

(5) $$d_{s}=\frac{\left|s_{1i}-s_{2j}\right|}{s_{2j}}$$

*d_p_* denotes the positional variation of storm cells, and *d_s_* represents the area change rate of storm cells, as expressed in Equations (4) and (5), respectively. *w*_1_ and *w*_2_ are the weight coefficients corresponding to *d_p_* and *d_s_*, respectively. $\left(\bar{x},\bar{y}\right)$ refers to the centroid coordinates of a single storm cell, and *S* is the storm cell area.

When calculating the cost function *C_ij_* between the i-th storm cell at time *t*_1_ and the j-th storm cell at the successive time *t*_2_, two constraints must be imposed. The angle between the displacement vector of matched storm cells at adjacent times and the instantaneous velocity vector of the storm cell is less than 90°, and the displacement speed of matched cells between successive times is no more than 60 km⋅h^−1^. These two constraints are expressed in Equations (6) and (7), respectively.

(6) $$[vx_{1i},vy_{1i}]•[{\bar{x}}_{2j}-{\bar{x}}_{1i},{\bar{y}}_{2j}-{\bar{y}}_{1i}]>0$$

(7) $$\frac{d_{p}}{t_{2}-t_{1}}\leq 60\;\mathrm{km}\cdot h^{-1}$$

[*vx*,*vy*] denotes the velocity vector of a storm cell. [*vx*_1*i*_,*vy*_1*i*_] represents the instantaneous velocity vector of the i-th storm cell at time *t*_1_, while $\left[{\bar{x}}_{2j}-{\bar{x}}_{1i},{\bar{y}}_{2j}-{\bar{y}}_{1i}\right]$ corresponds to the displacement vector between the i-th storm cell at time *t*_1_ and matched the j-th storm cell at the subsequent time *t*_2_.

Finally, the minimum value of cost function *C_ij_* indicates the optimal matching between the i-th storm cell at time *t*_1_ and the j-th storm cell at the subsequent time *t*_2_, meaning that these two storm cells correspond to the identical cell. Furthermore, we perform matching for all storm cells at successive time steps and integrate all optimal matching relationships. The historical moving trajectory of each storm cell can thereby be extracted, which realizes the full tracking of storm cell evolution over time.

### 3.4. Intensity Prediction

Storm cell intensity does not follow a simple linear trend throughout its evolutionary process, and it generally undergoes dynamic transitions among three states: intensification, weakening, and stable maintenance. Based on the intensity assessment presented in Section 3.2 and the historical tracking outcomes in Section 3.3, the Adaptive Damped Holt’s Method is employed in this study to forecast storm cell intensity. This model decomposes the intensity time series into level and trend components, which are mathematically defined by Equations (8) and (9), respectively.

*L*(*t*) = *αR*(*t*) + (1 − *α*)*L*(*t* − 1) + *ϕB*(*t* − 1) (8)

*B*(*t*) = *λ*[*L*(*t*) − *L*(*t* − 1)] + (1 − *λ*)*ϕB*(*t* − 1) (9)

In the above equations, *t* and *t* − 1 denote the current and previous time steps, respectively. Here, *α* represents the smoothing coefficient of the level component, and *λ* denotes the smoothing coefficient of the trend component, which are set to 0.6 and 0.3 in this study. The parameter *ϕ* is a damping factor for the trend component, which enables the gradual attenuation of long-term extrapolation trends and prevents excessive extrapolation when the evolutionary trend of storm cells is uncertain. Prior to prediction, real-time state recognition results are adopted to identify the historical state of storm cells, including intensification, weakening, and stable maintenance. The damping factor *ϕ* is dynamically adjusted accordingly to adapt to the nonlinear and stochastic evolutionary characteristics of storm intensity, and the specific calculation formula is presented in Equation (10).

(10) $$\left\{\begin{array}{cc} \phi =0.90 & (0.1\leq \left|k\right|<0{.2,R}^{2}\leq 0.7)\\ \phi =0.95 & (0.2\leq \left|k\right|<0{.5,R}^{2}\leq 0.7)\\ \phi =0.98 & (0.1\leq \left|k\right|<0{.2,R}^{2}\leq 0.7)\\ \phi =0 & \mathrm{Others} \end{array}\right.$$

*k* is the slope derived from linear regression of historical intensity time series, while the coefficient of determination *R*^2^ measures the goodness-of-fit between the regression model and observations. Larger *R*^2^ values correspond to superior model fitting performance.

Prior to prediction, we iteratively update the level component *L*(*t*) and trend component *B*(*t*) using historical intensity time series of storm cells. The storm cell intensity forecasting formula is given in Equation (11). The predicted intensity is jointly determined by *L*(*t*) and damped trend component *B*(*t*), and the trend term decays over time to eliminate unreasonable extrapolation.

(11) $$R(t+n)=L(t)+B(t)\sum_{\delta =1}^{n}\phi^{\delta }$$

### 3.5. Track Prediction

The Unscented Kalman Filter (UKF) is a recursive filtering algorithm for state estimation of nonlinear systems. It adopts the Unscented Transform (UT) to approximate nonlinear mappings without calculating Jacobian matrices, thus achieving superior adaptability to nonlinear systems. The core principle of UKF lies in selecting a set of sigma points to approximate the system state distribution. Nonlinear transformation is performed on these sigma points, and the mean and covariance of transformed points are calculated to estimate the system state and its covariance [24,25]. As expressed in Equation (12), 2n+1 sigma points are generally adopted to fit the probability distribution of state variables for an n-dimensional state vector.

(12) $$\left\{\begin{array}{l} \gamma_{k-1}^{\left(0\right)}=x_{k-1}\;\omega_{0}=\frac{\lambda }{n+\lambda }\\ \gamma_{k-1}^{\left(i\right)}=x_{k-1}+\sqrt{(n+\lambda {)P}_{k-1}}\;\omega_{i}=\frac{\lambda }{2n+\lambda }\;i=1\ldots n\\ \gamma_{k-1}^{\left(i+n\right)}=x_{k-1}-\sqrt{(n+\lambda {)P}_{k-1}}\;\omega_{i}=\frac{\lambda }{2n+\lambda }\;i=1\ldots n \end{array}\right.$$

$\gamma_{k-1}^{\left(i\right)}$ denotes the discrete distribution of sigma points at time step *k* − 1, and *ω_i_* represents the weight coefficient corresponding to each sigma point. *x_k_*_−1_ and *P_k_*_−1_ are the system state vector and covariance matrix at time step *k* − 1, respectively. *λ* is the scaling parameter, which is set to 1 in this study.

The UKF algorithm consists of two core stages: prediction and update. In the prediction stage, the system state at the current time step is inferred from the state information of the previous time step, which involves inherent uncertainty. The predictions of system state and covariance are expressed in Equations (14) and (15), respectively. *x_k_*_|*k*−1)_ and *P_k_*_|*k*−1)_ represent the state vector and covariance matrix at time step k derived from the k-1 time step, and ∑w denotes the process noise covariance matrix (∑w=diag(0.1,0.1,0.1,0.1,0.01)). Prior to prediction, as described in Equation (13), the state transition function *f*() is adopted to implement the nonlinear transformation of sigma points.

(13) $$x_{k\left|k-1\right.}=\sum_{i=0}^{2n}\omega_{i}{\bar{\gamma }}_{k-1}^{\left(i\right)}$$

(14) $$P_{k\left|k-1\right.}=\sum_{i=0}^{2n}\omega_{i}({\bar{\gamma }}_{k-1}^{\left(i\right)}-x_{k\left|k-1\right.}){\left({\bar{\gamma }}_{k-1}^{\left(i\right)}-x_{k\left|k-1\right.}\right)}^{T}+\sum w$$

(15) $${\bar{\gamma }}_{k\left|k-1\right.}^{\left(i\right)}=f(\gamma_{k-1}^{i})$$

The update stage corrects the system parameters using observational data to improve future prediction accuracy. The updating procedures for system state and covariance are illustrated in Equations (16)–(22). *Z_k_*_|*k*−1)_ denotes the observation vector at time step *k* predicted from the *k* − 1 state, *P_ZZ_* represents the measurement covariance, *P_XZ_* is the cross-covariance between the state vector and observation vector, and ∑v indicates the measurement noise covariance (∑v=diag(1.0,1.0)). Prior to the update operation, the predicted sigma points are nonlinearly transformed via the observation transition function *h*(), as defined in Equation (16).

(16) $${\hat{\gamma }}_{k\left|k-1\right.}^{\left(i\right)}=h({\bar{\gamma }}_{k\left|k-1\right.}^{\left(i\right)})$$

(17) $$Z_{k\left|k-1\right.}=\sum_{i=0}^{2n}\omega_{i}{\hat{\gamma }}_{k\left|k-1\right.}^{\left(i\right)}$$

(18) $$P_{\mathrm{ZZ}}=\sum_{i=0}^{2n}\omega_{i}({\hat{\gamma }}_{k\left|k-1\right.}^{\left(i\right)}-Z_{k\left|k-1\right.}){\left({\hat{\gamma }}_{k\left|k-1\right.}^{\left(i\right)}-Z_{k\left|k-1\right.}\right)}^{T}+\sum v$$

(19) $$P_{\mathrm{XZ}}=\sum_{i=0}^{2n}\omega_{i}({\bar{\gamma }}_{k-1}^{\left(i\right)}-x_{k\left|k-1\right.}){\left({\bar{\gamma }}_{k\left|k-1\right.}^{\left(i\right)}-Z_{k\left|k-1\right.}\right)}^{T}$$

*K_k_* = *P_XZ_*/*P_ZZ_* (20)

*x_k_* = *x_k_*_|*k*−1)_ + *K_k_*(*Z_k_* − *Z_k_*_|*k*−1_) (21)

*P_k_* = *P_k_*_−1_ + *K_k_P_ZZ_K_k_^T^* (22)

*K_k_* denotes the Kalman gain for system state updating and correction. *x_k_* and *P_k_* represent the final updated state vector and covariance matrix, respectively.

In this study, the UKF state vector is defined as a five-dimensional variable, which consists of the centroid coordinates (*Cx_k_*,*Cy_k_*), translational velocity *v_k_*, Heading angle *θ_k_*, and angular velocity *ψ_k_* of storm cells. The formulation of the prediction model is presented in Equation (23), and the UKF state vectors at adjacent time steps *k* − 1 and *k* satisfy the following temporal relationship.

(23) $$\left\{\begin{array}{l} Cx_{k}=Cx_{k-1}+v_{k}\cos(\theta_{k})\\ Cy_{k}=Cy_{k-1}+v_{k}\sin(\theta_{k})\\ \theta_{k}=\theta_{k-1}+\psi_{k}T\\ v_{k}=v_{k-1}\psi_{k}=\psi_{k-1} \end{array}\right.$$

The proposed method enables the prediction of future trajectories of storm cells. Storm cell spatial location prediction is essential for operational meteorological early warning systems. In this study, the instantaneous storm intensity at the current moment is adopted as the initial intensity *R*(*t*), and the area of the optimal external ellipse is defined as the initial storm cell spatial location *S*(*t*). The future storm cell spatial location *S*(*t* + *n*) is assumed to be linearly correlated with the storm intensity *R*(*t* + *n*), as formulated in Equation (24), where the linear coefficient *κ* = 1. By mapping the ellipse center to the predicted future motion trajectory, the spatial fall distribution of storm cells can be finally obtained.

(24) $$\frac{S(t+n)}{S(t)}=\kappa \frac{R(t+n)}{R(t)}$$

### 3.6. Method Evaluation

To quantitatively evaluate the prediction accuracy of storm cell spatial location, three statistical metrics, including POD, FAR, and CSI, are adopted to comprehensively assess the model performance. Specifically, the POD reflects the capability of correct prediction, the FAR quantifies the false prediction of unobserved events, and the CSI evaluates the overall forecasting performance. The mathematical expressions of the three metrics are defined in Equations (25)–(27). The definitions of true positive (TP), false positive (FP), and false negative (FN) samples are listed in Table 4. It should be emphasized that a predicted storm cell is regarded as TP if its spatial coverage and that of the corresponding observed storm cell satisfy the following two criteria: (1) the overlapping area accounts for no less than 20% of the area of the observed storm cell; (2) the centroid distance between the two storm cells is less than 20 km. In practical verification, higher POD and CSI values and a lower FAR value indicate superior prediction performance, and vice versa.

(25) $$\mathrm{POD}=\frac{\mathrm{TP}}{\mathrm{TP}+\mathrm{FN}}$$

(26) $$\mathrm{FAR}=\frac{\mathrm{FP}}{\mathrm{TP}+\mathrm{FP}}$$

(27) $$\mathrm{CSI}=\frac{\mathrm{TP}}{\mathrm{TP}+\mathrm{FN}+\mathrm{FP}}$$

To quantitatively evaluate the accuracy of storm intensity prediction, *MAE* is employed to assess the predictive performance, as defined in Equation (28). *P_i_* and *T_i_* denote the predicted intensity and the observed true intensity of the i-th storm cell, respectively. Smaller *MAE* value indicates better intensity prediction performance, while a larger value corresponds to lower prediction accuracy.

(28) $$\mathrm{MAE}=\frac{1}{n}\sum_{i=1}^{n}\left|P_{i}-T_{i}\right|$$

## 4. Application

### 4.1. Case 1: Severe Thunderstorm Wind Event, 5 August 2023

**Figure 6:**
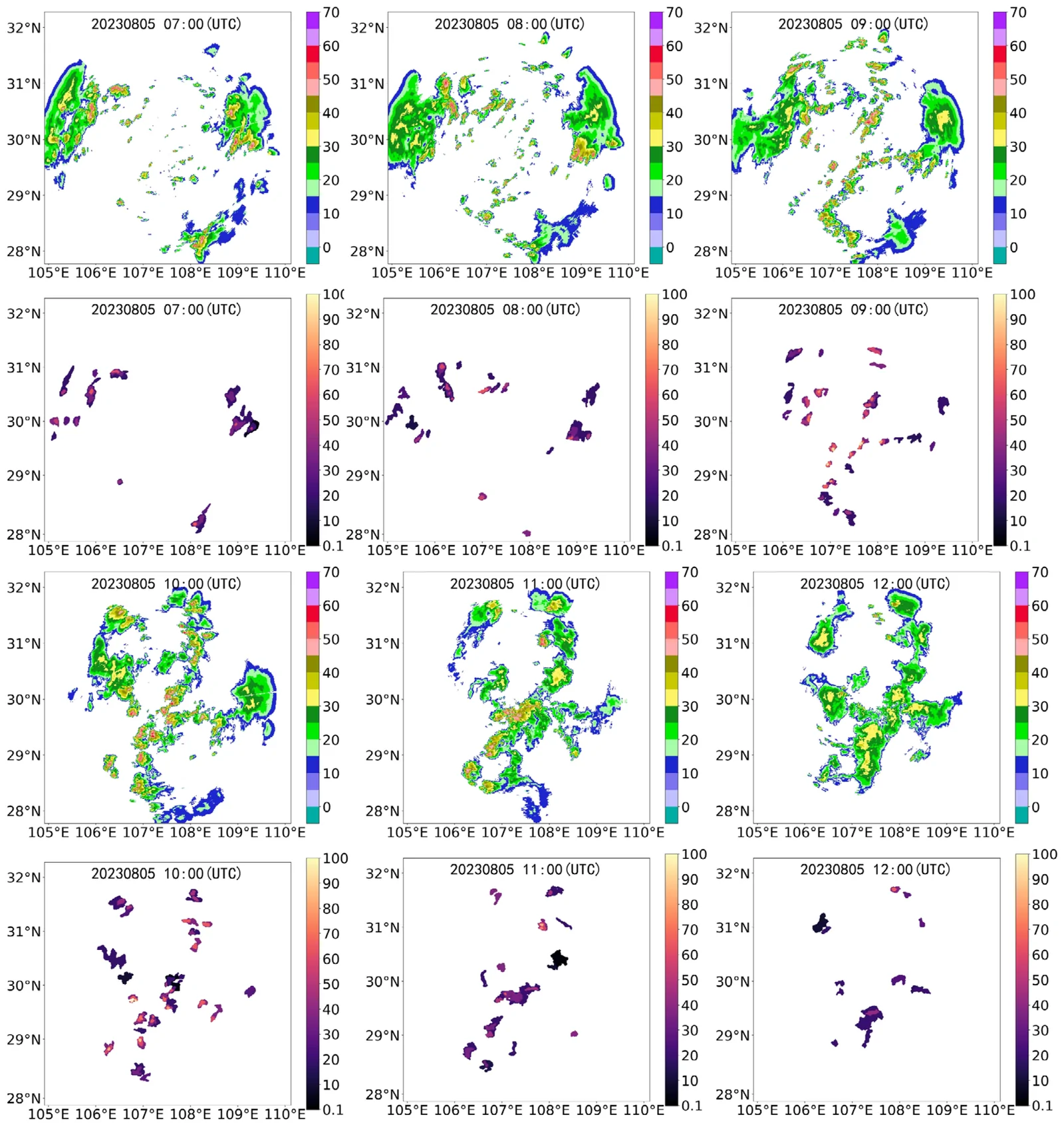
Radar reflectivity fields (CAPPI-3KM, range: −5dBZ–70dBZ) and storm cell intensity assessment results (range: 0.1–100) for the severe thunderstorm winds event in Chongqing on 5 August 2023.

A severe thunderstorm winds event occurred in Chongqing during 07:00–12:00 on 5 August 2023. During this episode, heavy rainfall was captured at 18 stations covering 10 districts, with a peak hourly rainfall of 64.8 mm recorded at Fuling Station at 11:00. Local gusts stronger than Grade 8 impacted 12 districts, and the maximum wind speed of 29.0 m/s was observed at Zhongxian Station at 09:00. The 3 km Constant Altitude Plan Position Indicator (CAPPI-3KM) radar reflectivity fields and storm cell intensity assessment results for this severe convective event are illustrated in Figure 6.

From 07:00 to 08:00, discrete severe convective cloud clusters developed over the northwestern and eastern regions of Chongqing while exhibiting an overall weakening trend. Conversely, numerous convective cells continuously initiated and rapidly intensified over central Chongqing, with radar reflectivity exceeding 50 dBZ at the cell centroids. During 09:00–10:00, isolated storm cells along the northeast–southwest corridor self-organized into a well-defined squall line that propagated eastward at a rapid speed. These storm cells gradually expanded and coalesced into a continuous band-shaped radar echo, and the intensity of convective cells over central Chongqing surpassed 90 during this stage.

After 10:00, the organized squall line gradually decayed and transitioned into stratiform precipitation clouds. Although sporadic storm cells persisted over central Chongqing, their intensities dropped below 60 and exhibited a sustained weakening tendency. All storm cells completely dissipated after 12:00, along with the gradual attenuation of precipitation, which denoted the termination of this severe convective event. Overall, the storm cell identification and intensity evaluation results are highly consistent with the spatiotemporal evolution of observed radar echoes, demonstrating the reliability and validity of the assessment scheme.

**Figure 7:**
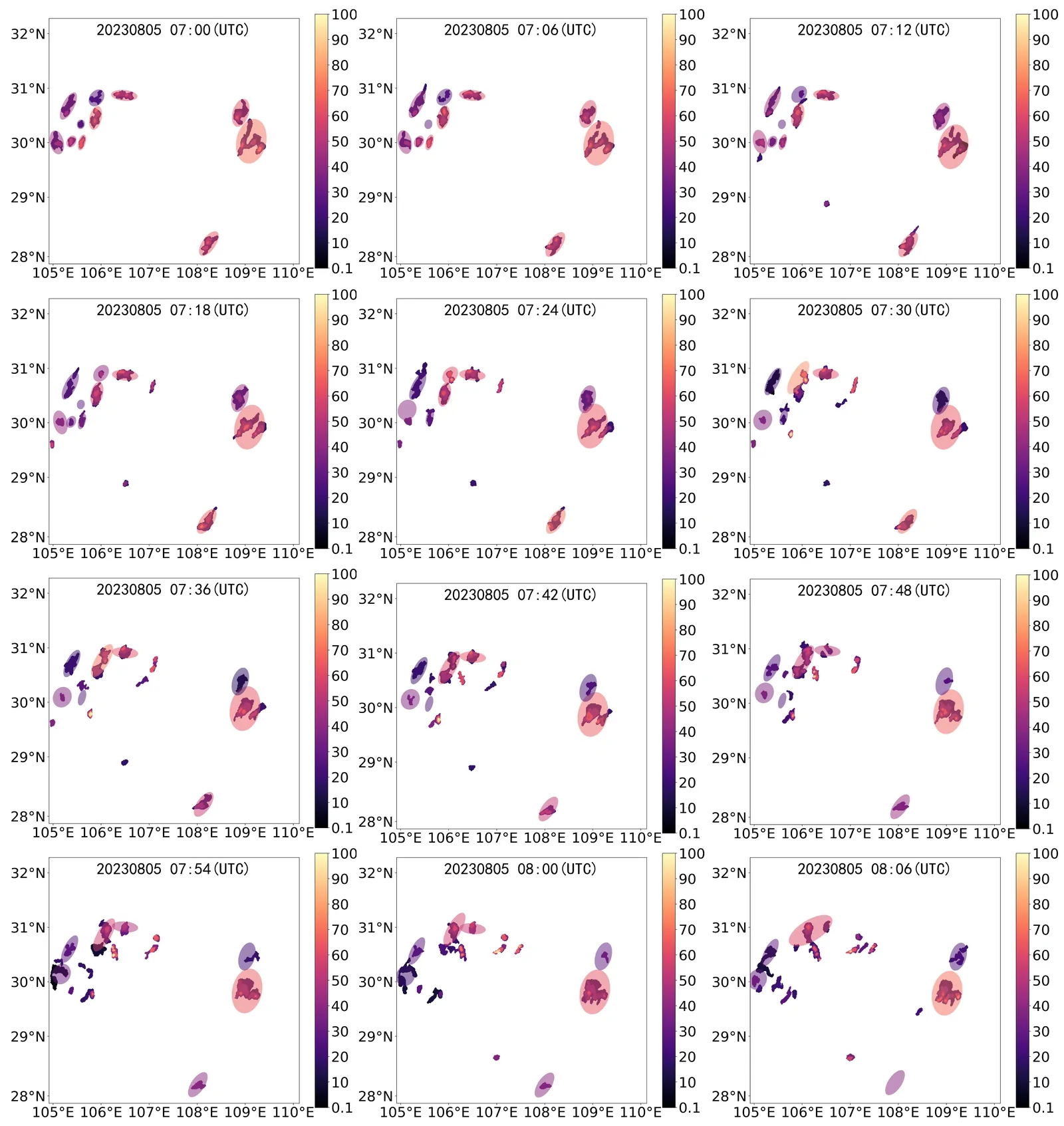
Comparison between predicted and observed thunderstorm winds for the subsequent 1 h forecast, initialized at 07:00 on 5 August 2023.

Figure 7 presents the comparative analysis between thunderstorm winds forecasts and corresponding observed spatial distributions. The forecasts were initialized at 07:00 on 5 August 2023, with outputs generated every 6 min over the subsequent 1-h forecast window. The elliptical contours in the figure denote the spatial coverage of forecast storm cells, and the fill color of each ellipse corresponds to the maximum intensity of the matched storm cell. Extreme intensity values are the primary concern in operational meteorological early warning services.

Comparisons of storm coverage forecasts indicate that the predicted storm areas well match observations at the initial forecast time. Within the 30-min forecast lead time, sporadic newly generated storm cells cause slight missing forecasts, while the overall prediction accuracy remains above 70%. At the 60-min lead time, abundant newly initiated convective cells lead to substantial missing forecasts, yet the forecast performance for large-scale storm systems remains acceptable. This characteristic is mainly attributed to the storm identification rule: when two forecast cell regions overlap, they are merged into a single storm object, and a new circumscribed ellipse is fitted to represent the integrated coverage. In terms of intensity prediction, the maximum intensity represented by forecast ellipses generally agrees with the central intensity of observed storm cells, and the bias between predicted and observed intensities gradually increases as the forecast lead time extends.

### 4.2. Case 2: Severe Thunderstorm Wind Event, 18 July 2025

From 06:00 to 11:00 on 18 July 2025, Chongqing experienced a severe thunderstorm winds event. Torrential rainfall was recorded at four precipitation stations across two districts during the event, with the peak hourly rainfall intensity of 65.2 mm·h^−1^ observed at Wanzhou Station at 08:00. Local gusts of Grade 8 or above were reported in 23 districts, and the maximum gust speed of 33.2 m/s was captured at Jiangjin Station at 07:00.

**Figure 8:**
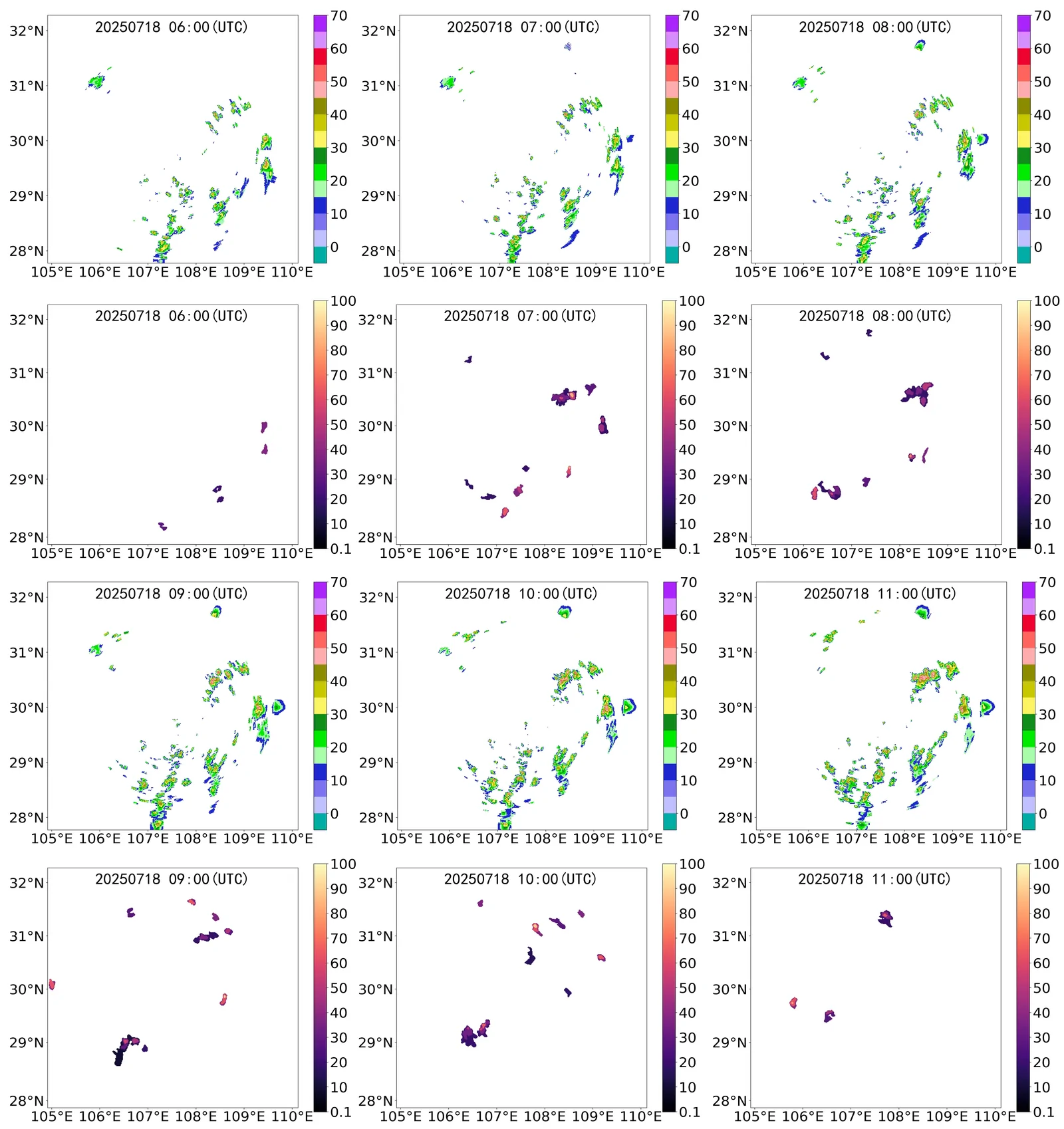
Radar reflectivity fields (CAPPI-3KM, range: −5 dBZ–70 dBZ) and storm cell intensity assessment results (range: 0.1–100) for the severe thunderstorm winds event in Chongqing on 18 July 2025.

The CAPPI-3KM radar reflectivity fields and storm cell intensity assessment results for this severe convective event are illustrated in Figure 8. At 06:00, convective cloud clusters over the southeastern Chongqing to northern Guizhou region developed rapidly and propagated northwestward, triggering the continuous genesis of numerous storm cells along the line. From 07:00 to 10:00, the scattered convective systems organized into a squall line, which steadily maintained along the northwest–southeast-oriented Yangtze River mainstream in Chongqing, with the radar reflectivity of individual storm centroids generally exceeding 50 dBZ. During 07:00–09:00, storm cells continuously increased in number and expanded in scale, forming a linear banded structure that persisted for nearly two hours, with the maximum estimated intensity of partial storm cells reaching above 90. During 09:00–10:00, the intensity of storm cells gradually weakened, with all cell intensity values dropping below 60. After 10:00, the squall line dissipated and transformed into stratiform stable precipitation cloud clusters, accompanied by a continuous reduction in precipitation intensity and the complete dissipation of all storm cells, marking the termination of this severe convective event. Overall, the identified storm cell characteristics and evaluated intensity exhibit a high consistency with the actual radar observations.

**Figure 9:**
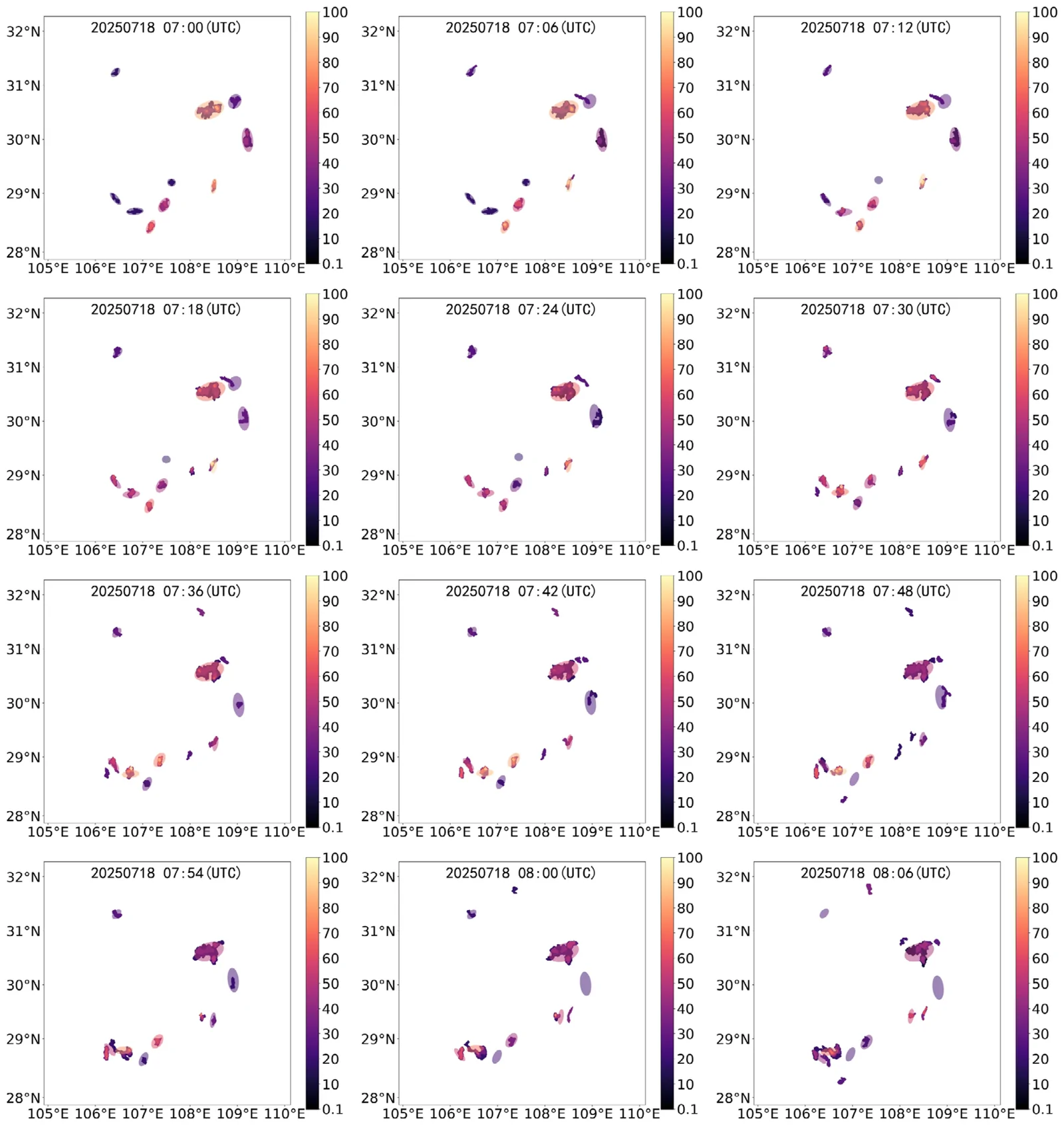
Comparison between predicted and observed thunderstorm winds for the subsequent 1-h forecast, initialized at 07:00 on 18 July 2025.

The comparison between predicted and observed storm cells is presented in Figure 9. Forecast fields were initialized at 07:00 on 5 August 2023, with model outputs produced at a 6-min interval throughout the subsequent 1-h forecast lead time. For pre-existing storm cells, the predicted coverage shows satisfactory agreement with radar observations. After the 30-min lead time, continuous initiation of new storm cells leads to a gradual rise in false alarm rate (FAR) and a decline in forecast accuracy. Nevertheless, the proposed method can reliably capture the spatial coverage of long-lived persistent storm cells. Additionally, small biases exist between predicted maximum central reflectivity of storm cells and corresponding observations, demonstrating that this approach is well suited for operational severe storm forecasting and early warning services.

## 5. Discussion

**Figure 10:**
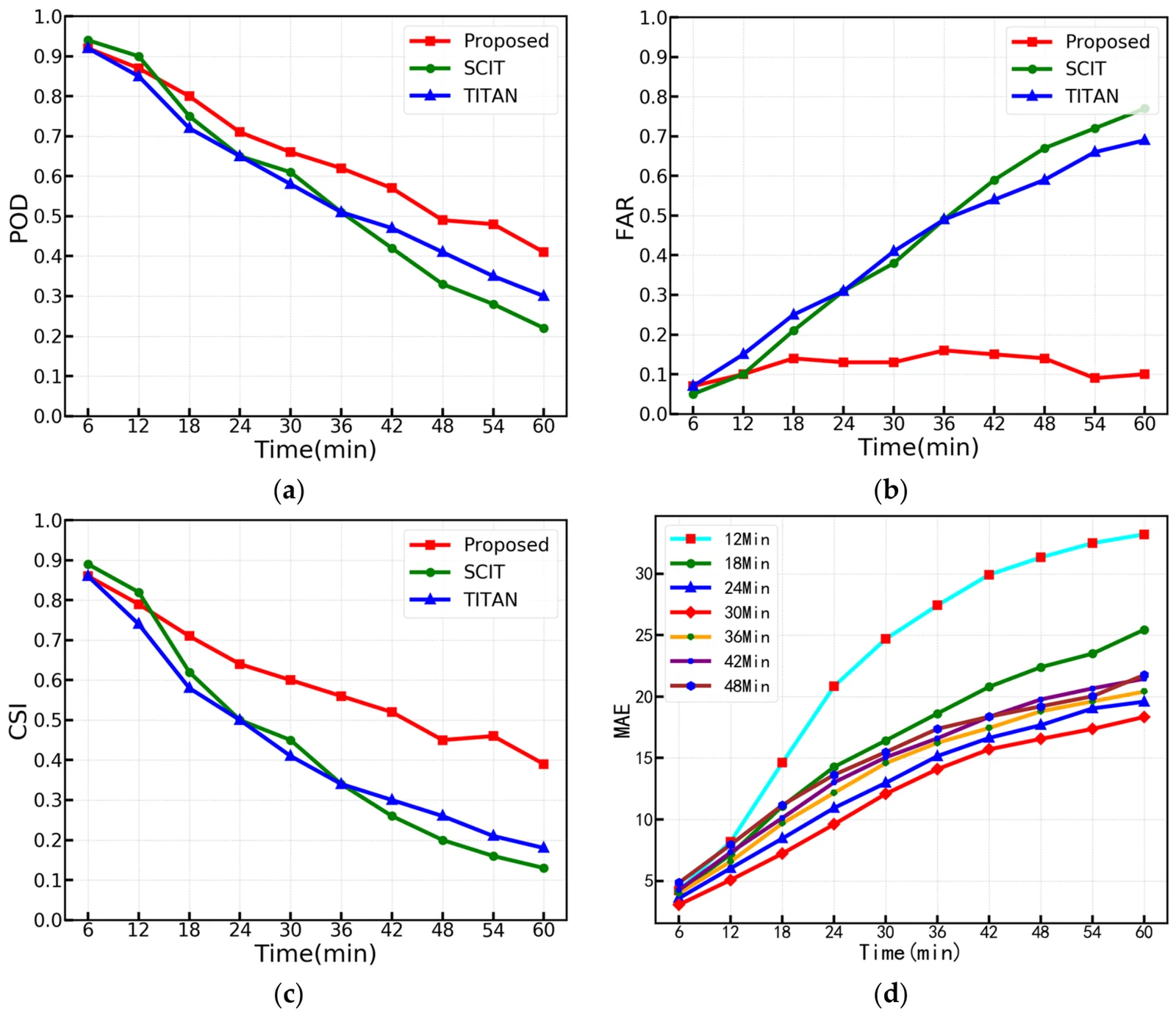
Evaluation of storm cell spatial location prediction and intensity prediction. (**a**) POD, (**b**) FAR, (**c**) CSI, (**d**) MAE.

To quantitatively verify the accuracy and reliability of the proposed storm cell prediction method, we adopt the evaluation metrics defined in Section 3.6 to systematically evaluate the spatial and intensity prediction performance of the testing cells. The testing dataset consists of 596 storm cells derived from 16 screened severe convective weather events, as listed in Table 2. Meanwhile, we conduct comparative evaluations against two widely used operational algorithms in China’s meteorological services, SCIT and TITAN, which were introduced in the Introduction (Section 1). Figure 10 shows the quantitative evaluation results of storm cell prediction: Figure 10a–c assess spatial location prediction accuracy, and Figure 10d evaluates intensity prediction performance.

Figure 10a shows the probability of detection (POD) for storm cell spatial forecast. The POD values of the proposed method, SCIT, and TITAN all gradually decrease with increasing forecast lead time, presenting a negative correlation with forecast duration. The POD values start at 92%, 94%, and 92% at the initial time, drop to 66%, 61%, and 58% at the 30-min lead time, and further decrease to 41%, 22%, and 30% at the 1-h lead time. The comparison reveals that the three methods yield similar POD within the first 12 min. After 12 min, the proposed method exhibits an increasingly larger advantage over SCIT and TITAN. SCIT and TITAN have comparable performance within the first 36 min, while the POD gap between TITAN and SCIT gradually widens beyond 36 min. The gradual degradation of POD arises from the accumulation of forecast errors and the formation of new storm cells. Spatial discrepancies between predicted and observed storm cells induced by early forecast biases tend to be amplified with increasing lead time, inevitably degrading the prediction accuracy at subsequent moments. Meanwhile, the unpredictable initiation of new storm cells also contributes to the decline in POD.

Figure 10b shows the false alarm rate (FAR) for storm cell spatial forecast. The FAR of the proposed method fluctuates slightly throughout the entire forecast period and consistently remains below 15%. In contrast, the FAR of both SCIT and TITAN gradually increases with increasing forecast lead time, showing a positive correlation with forecast duration. The proposed method exhibits a clear advantage in terms of FAR. This is mainly attributed to the storm cell matching strategy, which effectively suppresses false alarms: historical storm cells that fail to match any observed counterparts for three consecutive timestamps are considered dissipated and excluded from subsequent matching and evaluation procedures. This mechanism eliminates the interference of invalid samples and keeps the FAR at a low and stable level.

Figure 10c shows the critical success index (CSI) for storm cell spatial forecast. The CSI values of the proposed method, SCIT, and TITAN all gradually decrease with increasing forecast lead time, exhibiting a negative correlation with forecast duration. The CSI starts at 86%, 89%, and 86% at the initial time, drops to 60%, 45%, and 41% at the 30-min lead time, and further decreases to 39%, 13%, and 18% at the 1-h lead time. The comparison indicates that the proposed method achieves a remarkable advantage over SCIT and TITAN in terms of CSI. This benefit arises from two aspects. First, the advantage of the proposed method in POD continuously grows as the forecast lead time increases. Second, the FAR of the proposed method does not rise with lead time, which is distinctly superior to the linearly increasing FAR of SCIT and TITAN.

It should be noted that, unlike storm cell spatial forecast, storm cell intensity prediction is a newly developed component in this study, and hence comparative analyses with SCIT and TITAN are not available. Figure 10d illustrates the quantitative evaluation results of storm cell intensity prediction, where each curve corresponds to the model configured with historical intensity datasets of different time lengths. This study proposes an adaptive damped Holt-based intensity prediction method for storm cells in Section 3.4. The established model performs dynamic forecasting of future intensity evolution by learning temporal variations in historical storm intensity, and its prediction accuracy is highly sensitive to the time length of input historical samples. Comparative experiments demonstrate that the model driven by historical intensity data within the preceding 30 min achieves the optimal forecasting performance. Specifically, insufficient historical samples fail to fully capture the continuous evolutionary characteristics of storm intensity, while excessive time-series inputs lead to data redundancy and amplify temporal noise, thereby degrading model generalization and prediction stability. Overall, the MAE increases steadily with extended forecast lead time, indicating a growing discrepancy between predicted and observed storm intensity. The prediction accuracy exhibits a prominent negative linear correlation with forecast duration. For the optimal model with a 30-min historical input window, the MAE starts at 3 at the initial forecast time, rises to 12 at the 30-min lead time, and further increases to 18 for the 1-h forecast. From the perspective of operational meteorological application, the prediction errors within the 1-h forecast window are generally acceptable, which satisfies the operational requirements for nowcasting and early warning of severe convective weather.

However, this study has several limitations. First, only radar reflectivity products are adopted in our analysis. Dual-polarization radar parameters carry rich information regarding hydrometeor phases and microphysical processes and show great application potential. Integrating fused dual-polarization products into storm cell identification and trend forecasting therefore represents an important direction for future research. Second, all samples are derived from the Chongqing radar network, which limits the generalization ability of the proposed model. Minor recalibration of model parameters may be required when applying this method to regions with markedly different terrain, such as plains and coastal areas. Additional experiments using multi-terrain datasets will be carried out in future work.

## 6. Conclusions

Based on CAPPI reflectivity features derived from networked weather radars, this study proposes an integrated algorithm for storm cell identification, intensity evaluation, tracking, and nowcasting, which can be applied to operational forecasting and early warning of severe thunderstorm winds. Digital image processing techniques are adopted to automatically detect three-dimensional storm cells, enabling intuitive and efficient extraction of spatial distribution of convective storms. Using the evolutionary trends of storm structural variables throughout the whole lifecycle, including cell area, vertical extent, and centroid height, a fuzzy logic framework is developed to evaluate storm intensity, which improves the objectivity and reliability of intensity classification. For storm tracking, a minimum cost function is constructed to match optimal storm cells between consecutive timestamps, realizing continuous trajectory tracking of individual storm cells. In the nowcasting module, the Unscented Kalman Filter is utilized to extrapolate storm moving paths, while the Adaptive Damped Holt’s method predicts storm intensity. Combinedly, this system generates 6-min interval storm nowcasting products covering a 0–60 min lead time.

Compared with the operationally mature SCIT and TITAN algorithms widely adopted in China’s meteorological services, the proposed method exhibits progressively superior performance in storm cell spatial prediction within a 60-min forecast window, with its advantages continuously amplified as the forecast lead time increases. Quantitative verification results indicate that although the POD and CSI of the proposed method decrease linearly with the extension of forecast duration, the spatial prediction accuracy remains above 60% at the 30-min lead time and approximately 40% at the 60-min lead time. Meanwhile, the FAR is stably controlled within 15% throughout the entire forecast period, demonstrating the reliable application potential of the proposed method in routine meteorological operations. Furthermore, the newly integrated intensity prediction module effectively compensates for the functional deficiencies of traditional tracking algorithms and better meets the operational requirements of severe convective weather early warning. The mean absolute error (MAE) of storm intensity prediction is less than 12 at the 30-min lead time and below 18 at the 60-min lead time, which fully verifies that the developed algorithm can satisfy the operational demands for severe thunderstorm wind warning services.

## Figures and Tables

**Table 1:** Overview of Radar Stations in the Chongqing Networked Radar Observation System.

Site	Band	Dual-Polarization	Altitude	Max Blockage Azimuth	Sum Blockage Azimuth
Chongqing	S-band	Original	210 m	<1°	<2°
Wanzhou	S-band	Upgrade	1066 m	<1°	<2°
Fuling	S-band	Original	895 m	<1°	<2°
Yongchuan	S-band	Original	640 m	<1°	<2°
Qianjiang	S-band	Upgrade	1700 m	<1°	<2°

**Table 2:** Datasets of Severe Thunderstorm Wind Events Collected in Chongqing From 2023 to 2025.

Storm Type	Events	Storm Cells	Training Cells	Testing Cells
City-wide	12	1142	568 (6 events)	474 (6 events)
Local	20	217	95 (10 events)	122 (10 events)

**Table 3:** Parameter settings of the trapezoidal, sigmoid, and Gaussian membership functions.

Features	Membership Functions	Storm Cell	Cell Core	Cell Centroid
horizontal projected area	$\mathrm{Trap(x)}=\left\{\begin{array}{cc} 0 & x<T_{1}\\ \frac{x-T_{1}}{T_{2}-T_{1}} & T_{1}<x<T_{2}\\ 1 & T_{2}<x<T_{3}\\ \frac{T_{4}-x}{T_{4}-T_{3}} & T_{3}<x<T_{4}\\ 0 & x>T_{4} \end{array}\right.$	*T*_1_ = 10 *T*_2_ = 50 *T*_3_ = 150 *T*_4_ = 300	*T*_1_ = 10 *T*_2_ = 30 *T*_3_ = 100 *T*_4_ = 200	*T*_1_ = 5 *T*_2_ = 10 *T*_3_ = 50 *T*_4_ = 100
vertical height	$S\mathrm{ig}(x)=\frac{1}{1+e^{-k(x-\eta )}}$	*η* = 3.5 *k* = 5	*η* = 2.5 *k* = 5	*η* = 1.5 *k* = 4
centroid height	$\mathrm{Gauss}(x)=\frac{e^{-\left(x-\mu \right)2}}{2\sigma^{2}}$	*μ* = 7 *σ* = 1.5	*μ*= 7 *σ*= 2.3	*μ* = 7 *σ*= 3.0

**Table 4:** Confusion Matrix.

Index	Prediction	True
TP	1	1
FN	0	1
FP	1	0
TN	0	0

## Data Availability

The data and source code supporting this study are available from the corresponding author upon reasonable request. Public redistribution of radar data needs permission from the data provider and compliance with its data sharing rules. As additional research using the same dataset is still in progress, we cannot release the data to a public DOI repository currently.

## References

[1] Shield S.A., Houston A.L. (2024). A Comparison of Thunderstorm Identification Methods. J. Atmos. Ocean. Technol..

[2] Holmes J., Forristall G., Mcconochie J. Dynamic response of structures to thunderstorm winds. Proceedings of the 10th Americas Conference on Wind Engineering (ACWE 2005).

[3] Dixon M., Wiener G. (1993). TITAN: Thunderstorm Identification, Tracking, Analysis, and Nowcasting—A Radar-based Methodology. J. Atmos. Ocean. Technol..

[4] Voormansik T., Rossi P.J., Moisseev D., Tanilsoo T., Post P. (2017). Thunderstorm hail and lightning detection parameters based on dual-polarization Doppler weather radar data. Meteorol. Appl..

[5] Johnson J.T., Mackeen P.L., Witt A., Mitchell E.D.W., Stumpf G.J., Eilts M.D., Thomas K.W. (1998). The Storm Cell Identification and Tracking Algorithm: An Enhanced WSR-88D Algorithm. Wea Forecast..

[6] Han L., Fu S.X., Zhao L.F., Zheng Y., Wang H., Lin Y. (2009). 3D Convective Storm Identification, Tracking, and Forecasting—An Enhanced TITAN Algorithm. J. Atmos. Ocean. Technol..

[7] Horn B.K.P., Schunck B.G. (1982). Determining Optical Flow. Artif. Intell..

[8] Lucas B.D., Kanade T. An Iterative Image Registration Technique with an Application to Stereo Vision. Proceedings of the 7th International Joint Conference on Artificial Intelligence (IJCAI ’81).

[9] Farneback G. Very High Accuracy Velocity Estimation Using Orientation Tensors, Parametric Motion, and Simultaneous Segmentation of the Motion Field. Proceedings of the Eighth IEEE International Conference on Computer Vision (ICCV 2001).

[10] Farnebck G. (2002). Polynomial Expansion for Orientation and Motion Estimation. Doctoral Dissertation.

[11] Weinzaepfel P., Revaud J., Harchaoui Z., Schmid C. DeepFlow: Large Displacement Optical Flow with Deep Matching. Proceedings of the 2013 IEEE International Conference on Computer Vision (ICCV).

[12] Wulff J., Black M.J. Efficient sparse-to-dense optical flow estimation using a learned basis and layers. Proceedings of the 2015 IEEE Conference on Computer Vision and Pattern Recognition (CVPR).

[13] Lecun Y., Bottou L., Bengio Y., Haffner P. (1998). Gradient-based learning applied to document recognition. Proc. IEEE.

[14] He K., Zhang X., Ren S., Sun J. Deep Residual Learning for Image Recognition. Proceedings of the 2016 IEEE Conference on Computer Vision and Pattern Recognition (CVPR).

[15] Siegelmann H.T. (1995). Recurrent Neural Networks. Computer Science Today.

[16] Hochreiter S., Schmidhuber J. (1997). Long Short-Term Memory. Neural Comput..

[17] Chung J., Gulcehre C., Cho K.H., Bengio Y. (2014). Empirical Evaluation of Gated Recurrent Neural Networks on Sequence Modeling. arXiv.

[18] Wang Y., Long M., Wang J., Gao Z., Yu P.S. (2017). PredRNN: Recurrent neural networks for predictive learning using spatiotemporal LSTMs. Proceedings of the 31st International Conference on Neural Information Processing Systems (NIPS'17), Long Beach, CA, USA, 4–9 December 2017.

[19] Snderby C.K., Espeholt L., Heek J., Dehghani M., Oliver A., Salimans T., Agrawal S., Hickey J., Kalchbrenner N. (2020). MetNet: A Neural Weather Model for Precipitation Forecasting. arXiv.

[20] Geng H., Wu F., Zhuang X., Geng L., Xie B., Shi Z. (2024). The MS-RadarFormer: A Transformer-Based Multi-Scale Deep Learning Model for Radar Echo Extrapolation. Remote Sens..

[21] Liu Y., Zhang H., Chen L. (2022). Spatiotemporal Transformer Network for Radar Echo Nowcasting. Remote Sens..

[22] Chen X., Zhou B. (2026). Event-Guided Spatiotemporal Transformer with Conditional Diffusion Refinement for High-Intensity Precipitation Nowcasting. Remote Sens..

[23] Suzuki S., Be K. (1985). Topological structural analysis of digitized binary images by border following. Comput. Vis. Graph. Image Process..

[24] Wan E., van der Merwe R. (2002). The Unscented Kalman Filter. Kalman Filtering and Neural Networks.

[25] Cheng S., Mao Y. (2009). Iterated unscented Kalman filter. J. Data Acquis. Process..

